# High diluted molecules and gene expression

**DOI:** 10.3389/fphar.2014.00183

**Published:** 2014-08-14

**Authors:** Salvatore Chirumbolo

**Affiliations:** University Laboratories for Medical Research (LURM)-Medicine D, Department of Medicine, University of VeronaVerona, Italy

**Keywords:** gelsemine, *Gelsemium*, *Gelsemium sempervirens*, homeopathy, biases, microarray gene expression

Two recently published papers reported that water/ethanol dilutions of *Gelsemium sempervirens* Ait. extracts could modify gene expression on *in vitro* human neuronal cells (Marzotto et al., 2014; Olioso et al., 2014). Both papers were performed with the purpose to demonstrate cellular activity of *G. sempervirens* dilutions, on suited standardized cultures, and confirm the behavioral evidence previously reported elsewhere (Magnani et al., 2010). The authors used two models for their experimental setting, namely SH-SY5Y and CCL-127 or IMR32 neuroblastoma cell lines (Marzotto et al., 2014; Olioso et al., 2014). After an oligonucleotide microarray, the authors selected less than 10 genes for RT-PCR to be significantly expressed following *Gelsemium* treatment (Marzotto et al., 2014; Olioso et al., 2014). However, using human neuronal cell line to elucidate results obtained with mouse models appears quite disputable. The search for an involvement of neural genes related to anxiety/depression or mood disorders is biased by the expression of human genes having no orthologs/homologs in mice, where the authors reported evidence about *Gelsemium* action on behavioral tests in animal anxiety models. For example, the gene baculoviral IAP repeat containing 8 (BIRC 8) has no homologs in mice (only ortholog genes in *Pan troglodytes*), olfactory gene OR4X1 is not expressed (absent) in mouse, gene C1ORF167 appears to have a non-characterized ortholog gene LOC102634746 in mouse, certainly not matching the research purpose to relate olfactory gene to the behavioral test. Furthermore, some genes indicated to be downregulated by *Gelsemium 2c*, should not be expressed by neuronal cells (e.g. CD163, MPO, C8B, LST1, TREM2, notoriously expressed in immune cell). Both papers (Marzotto et al., 2014; Olioso et al., 2014) represent two interesting reports about gene expression microarray in homeopathy or ethnopharmacology, yet they deserves many comments about experimental setting and performance.

Preparations of herbal dilutions was carried out starting from a *G. sempervirens* “mother tincture” from Boiron and a gelsemine content of 6.5 × 10^−4^M was reported (Marzotto et al., 2014). Yet concentration of gelsemine was not assessed, as it was solely calculated on previous spectrometry investigations (Magnani et al., 2010) and new preparations, from ethanol draw extracts, were not further quantified by analytical chemistry. Although the authors reported UV-Vis spectra of *G. sempervirens*, which showed a peak at 250 nm caused by contaminating millimolar ethanol in *Gelsemium 2c*, they did not calculate any active principle dose in the tested solutions (Marzotto et al., 2014). Therefore, the authors tested a complex mixture of *G. sempervirens* extract, containing at least about 0.154 mM EtOH at 2c, if dilutions were conducted exactly, yet an alcoholic draw extract whose active principles were not identified for the investigation setting (Jin et al., 2014). The theoretical ethanol concentration evaluable by reading Marzotto's paper might reach actually 50 mM at *Gelsemium 2c* (Marzotto et al., 2014). A sub-millimolar dose of EtOH, close to 0.2 mM ethanol, is able to affect cell activity as BrdU incorporation, DNA fragmentation increase and LDH release, although data refer to HepG2 cell line at 24 h incubation with EtOH (Castañeda and Kinne, 2000). A strong apoptotic induction was related to ethanol doses as low as 100 mM for 24 h (Do et al., 2013) but 1.0 mM EtOH induce latent apoptosis in a cell line system and modifies expression of genes not necessarily involved in alcohol metabolism (Castaneda et al., 2007; Kupfer et al., 2013). Concentration of EtOH, set at 30% v/v, faded out to 0.003% in tested dilutions but the authors did not clarify how much for each centesimal dilution in the Methods section; the reader might trust the supposition that each centesimal dilution has about 50 mM EtOH. One paper reported that *G. sempervirens* 2c was prepared by diluting 100 times into simple distilled water a MT from Boiron (30 to 0.3% ethanol) to reach 1c and further 100 times (0.3 to 0.03% ethanol) to 2c (Marzotto et al., 2014). This may correspond to an ethanol concentration of about 50 mM at 0.3% EtOH. In Marzotto et al., more than 87% genes were downregulated, suggesting probably for a noxious action on cell function (Marzotto et al., 2014). Furthermore, the authors did not specify whether *G. sempervirens* preparations and serially diluted 30% alcohol/water solutions, were made under a laminar flow hood or provided sterile disposable plastic ware. This should lead to the conclusion that EtOH/water dilutions may not be endotoxin-free. To prevent bacterial contamination of the tested solutions, a Limulus amebocyte lysate test (LAL test) should be performed. Lipopolysaccharide can activate neuroblastoma genes (Nitta et al., 1994) and, if present as contaminant in herbal medicine, may affect gene microarray (Chang et al., 2012).

Finally the authors should have selected an array reporting a panoply of genes involved in the molecular mechanism they discussed about behavioral test (Magnani et al., 2010). Gene array profile of expression following 24 h incubation with *G. sempervirens* 2c, showed down-regulation of 49 genes, namely 87.5% gene array. Many gene products, listed in the expression profile of 56 genes array, such as LOC154872, KIAA0825, LOC150763, C1orf167, have not been identified (e.g. LOC644065) or are long intergenic non-coding protein RNA (C21orf24). Some genes are not reported/coded in the human genome, for example genes affected by *Gelsemium 2c*, such as DDl1, are bacterial genes, notoriously absent/non expressed in Homo sapiens: probably is a misprint (delta-like 1 (DLL1) instead of D-alanine-D-alanine ligase (DDl1) (Olioso et al., 2014), while other are exclusively expressed in humans, such as OR5C1 (olfactory receptor 5C1) while mouse has an olfactory receptor 368 gene on chromosome 2. Both papers did not show any exciting and significant action by very high diluted *G. sempervirens* solutions, contrarily to the conclusive remarks reported elsewhere (Magnani et al., 2010). The progression of the molecular assay revolution currently relies on the ability to efficiently and accurately offer multiplex detection and characterization for a variety of genes. Microarray analysis has the capability to offer robust multiplex detection but has just started to enter the research in high diluted substances. Although the use of microarrays to generate gene expression data has become routine, applications pertinent to high dilution pharmacology continue to rapidly expand. Further insights are needed to fix biases and misinterpretations leading to misunderstanding the effect of *G. sempervirens* raw extract respect to single active components.

## Conflict of interest statement

The author declares that the research was conducted in the absence of any commercial or financial relationships that could be construed as a potential conflict of interest.
